# Sleep Extension in Short Sleepers: An Evaluation of Feasibility and Effectiveness for Weight Management and Cardiometabolic Disease Prevention

**DOI:** 10.3389/fendo.2018.00392

**Published:** 2018-07-18

**Authors:** Theresa M. Pizinger, Brooke Aggarwal, Marie-Pierre St-Onge

**Affiliations:** ^1^Division of Endocrinology, Department of Medicine, New York Obesity Nutrition Research Center, Columbia University Medical Center, New York, NY, United States; ^2^Division of Cardiology, Department of Medicine, Columbia University Medical Center, New York, NY, United States; ^3^Institute of Human Nutrition, Columbia University Medical Center, New York, NY, United States

**Keywords:** sleep extension, cardiometabolic, health, sleep duration, prevention

## Abstract

Sleep duration has become increasingly recognized as an important influencer of health. Epidemiologic and observational studies have shown associations between short sleep duration and increased risk for chronic cardiometabolic disorders, including obesity, type 2 diabetes, and cardiovascular disease. These associations have led to investigations into the potential causal pathways through which short sleep may increase risk for these disorders. Clinical intervention studies have demonstrated that restricting sleep in normal sleepers has adverse health effects, including insulin resistance, and increased blood pressure. The totality of evidence points to negative health effects of short sleep and the recognition of sleep as a lifestyle behavior that may be targeted for disease prevention. It is well established that consistent, adequate sleep is associated with the lowest risk of obesity and cardiometabolic disorders. Yet, it is unclear whether increasing sleep in short sleepers can improve health. In today's society, it is common for individuals to deprive themselves of sleep during the work week, with the intent to sleep longer during the weekend, or have “catch-up sleep.” Studies that have examined the health effects of extended sleep, post-sleep restriction, revealed some improvements in health outcomes. However, it is uncertain whether the improvements observed with catch-up sleep are sufficient to reverse the negative health effects of constant sleep restriction. Few intervention studies have been undertaken to determine whether extending sleep, long-term, in short sleepers is feasible and whether it can reduce the disease risk burden associated with short sleep duration. The purpose of this review is to highlight these studies and evaluate information related to the impact of sleep extension on risk factors for chronic cardiometabolic disorders. We discuss limitations of current research, including variability in participant characteristics and the extent to which sleep behaviors are modified and monitored. Although the evidence-base for benefits of sleep extension is still in the early stages, studies to date indicate that prolonging sleep, in short sleepers, may improve cardiometabolic risk. Finally, our review calls attention to areas that require further study and for larger scale studies of behavior modification to establish the health effects of sleep extension in short sleepers.

## Introduction

Sleep duration has become increasingly acknowledged as an important factor in overall health status, and sleep deficiency has begun to be recognized as a potential modifiable risk factor for certain chronic conditions. Current research has shown that short sleep duration (SSD), defined as <7 h/night, is associated with an increased risk of obesity, metabolic disorders, and CVD (1–4). This association may be U-shaped, however, as there is also evidence that longer sleep duration, >9 h/night, is associated with adverse health effects (5). However, in today's “24/7" society, short sleep duration is more prevalent that long sleep duration. According to data from the National Health Interview Survey, the prevalence of very short sleep (<5 h) and short sleep (5–6 h) has increased from 1.7 to 2.4% and 19.7 to 26.7%, respectively, from 1977 to 2009 (6). Concurrently, long sleep (>9 h) decreased from 11.6 to 7.8% (6). This rise in the prevalence of short sleepers has permitted epidemiological evaluations of the influence of sleep duration on cardiometabolic risk factors. The data from these studies suggest a relation between sleep duration and obesity, hypertension, type 2 diabetes, and overall mortality (7, 8). These results have led to intervention studies to further examine the causal implications of these findings.

There are several mechanisms by which SSD may be related to obesity: metabolic changes affecting appetite-regulating hormones (9); low physical activity; and increased food intake without comparable change in energy expenditure (10, 11). Leptin is an adipocyte derived hormone that plays in important role in energy homeostasis (12). Its effect on energy balance regulation and SSD has been extensively studied, but with mixed results (13–15). Multiple clinical studies have shown that sleep restriction, in healthy, normal sleepers (7–9 h), leads to increases in daily energy intake (16–18) sufficient to lead to weight gain if sustained over time (18). On the other hand, there are conflicting reports regarding the impact of sleep restriction on energy expenditure (11, 17, 18). Nonetheless, it seems that any change in energy expenditure due to sleep restriction is either insufficient to offset the increase in energy intake or contributes to the positive energy balance.

Increased blood pressure (BP) and inflammatory markers, such as interleukin-6 (IL-6), are associated with systemic inflammation and decreased cardiovascular health (19). There are several clinical intervention studies examining the impact of sleep restriction, for periods ranging from a few days to a few weeks, on cardiometabolic risk factors (2–4, 20). When examining metabolic health, studies have demonstrated that sleep restriction reduces whole body insulin sensitivity and insulin resistance at a cellular level (21, 22). However, evidence linking SSD to adverse cardiometabolic risk does not de facto imply a benefit of sleep extension on health outcomes. In this review, we describe the cardiometabolic effects of sleep restriction, and we highlight and critically evaluate current interventions that aim to extend the duration of sleep in short-sleepers as a means of improving cardiometabolic risk factors. These studies are summarized in Table 1. We also address opportunities for future research in this area.

**Table 1:** Summary of clinical studies included in the present review that investigated the health effects of sleep extension.

**Study Ref**	**Intervention**	**Participants**	**Duration (Weeks)**	**Methods**	**Sleep as an outcome**	**Intervention group results**
Logue et al. (23)	Weekly group counseling sessions	25 overweight and obese adults	12	Group 1: diet and exercise counseling Group 2: diet and exercise counseling plus sleep education starting week 4	No Primary outcome: weight loss	Group 2 lost more weight compared to Group 1 (change of −5 vs. −2%) No difference in sleep quality
Tasali et al. (24)	1 individual counseling session at baseline to extend sleep and prescribe a sleep schedule	10 overweight adults	2	All subjects: increase TIB to 8.5 h/night, monitored with actigraphy No comparison group	Yes Extending sleep will improve appetite and decrease cravings	14% decrease in overall appetite and a 62% decrease in the desire for sweet and salty foods
Al Khatib et al. (25)	1 individual counseling session at baseline to extend sleep and prescribe a sleep schedule	41 healthy, normal weight adults	4	Sleep extension group: extend sleep by 1–1.5 h/night, monitored with actigraphy Control group	Yes Extending sleep will improve weight maintenance and cardiometabolic health	Reduced intake of free sugars (−9.6 g) compared baseline
Haack et al. (26)	Both groups received sleep hygiene information at baseline Extension group was prescribed individualized sleep schedules	22 adults with prehypertension or type 1 hypertension	6	Sleep extension group: extend sleep by 1 h/night, monitored with actigraphy Control group	Yes Extending sleep lowers BP	Decreased average systolic and diastolic beat-to-beat BP from baseline to endpoint (14 ± 3 vs. 8 ± 3 mmHg)
McGrath et al. (27)	Weekly individual sleep counseling	134 adults with elevated BP	8	Sleep intervention group: 60 min counseling session using Sleepio Standard care group	No Primary outcome: reduce mean 24 h BP	Reported improved sleep quality, no changes in BP
Leproult et al. (28)	1 individual counseling session at baseline to extend sleep and prescribe a sleep schedule	16 healthy adults	6	All subjects: extend sleep by 1 h/night, verified by actigraphy No comparison group	Yes Extending sleep will improve metabolic health	Improved insulin-to-glucose ratios

## Endocrine and cardiometabolic effects of recovery sleep after sleep restriction

In today's society, it is common for individuals to decrease their sleep during the work week, and to attempt to “catch-up” by sleeping longer on the weekends. It is reported that about 56% of Americans sleep less during the work week compared to non-work days (29). Several clinical trials have reproduced this scenario to understand the health effects of short term sleep recovery. Van Leeuwen et al. (30) tested the effects of sleep restriction and subsequent recovery sleep on glucose, leptin, and satiety in healthy men. Participants were sleep restricted for 5 days with a time in bed (TIB) of 4 h/night followed by a 2-day sleep recovery of 8 h TIB. Leptin levels were elevated during sleep restriction and remained elevated during the recovery period, compared to baseline, but no differences were found in subjective satiety measures throughout the experiment in both groups. It is possible that leptin remained elevated because an 8 h TIB was not an adequate amount of time to allow sufficient recovery sleep after 5 nights of <4 h sleep (31). Faraut et al. (31) investigated the effects that napping and recovery sleep had on the immune and inflammatory systems of healthy young men. Participants were restricted to 2 h TIB for one night followed by either an 8 h recovery night, a 30-min nap mid-day plus an 8 h recovery night, or a 10 h recovery night. The control group slept 3 consecutive nights of 8 h, with no changes in immune or inflammatory parameters throughout the experiment. Leukocyte counts increased in all sleep restricted groups compared to baseline, but this increase persisted in the 8 h recovery condition, while the numbers decreased in the nap + 8 h recovery and the 10 h recovery conditions. There was only a significant increase in myeloperoxidases in the sleep restriction +8 h recovery group; this increase was significant during both the restriction and recovery periods compared to baseline. The control and other 2 sleep restriction groups experienced no changes in inflammatory markers.

Spiegel et al. (32) used a more appropriate recovery sleep episode of 12 h TIB for 7 nights after participants were sleep restricted for 6 nights to 4 h TIB. In this group of healthy men, leptin levels during sleep restriction were 19% lower compared to the sleep recovery phase, suggesting that increasing sleep, following a period of drastically restricted sleep, may reverse the potential negative effects of short sleep on leptin levels. However, baseline leptin levels were not measured, and it is therefore unclear whether recovery sleep returned leptin levels to their baseline state or increased leptin levels. Difference between these two studies may have been due to the different lengths of the study or the duration of the recovery sleep period. The study by Spiegel et al. (32) had longer restriction and recovery phases than that of Van Leeuwen et al. (30), possibly allowing leptin to adapt to these changes in sleep. Another study (13) incrementally restricted sleep in 14 women for 4 nights, resulting in a decrease in leptin levels, with accompanying increases in energy intake and body weight. After 2 nights of recovery sleep, averaging 9.35 h/night, all of these parameters had returned to baseline.

In addition to energy balance outcomes, studies have also examined cardiometabolic risk factors in response to sleep extension. Van Leeuwen et al. (30) observed increases in fasting insulin and insulin-to-glucose ratio after sleep restriction, with both returning to baseline after the 2-night recovery period. Pejovic et al. (33) observed increases in 24 h plasma IL-6 levels following 6 nights of 6 h TIB. These levels returned to baseline after 3 nights of 10 h recovery sleep.

All of the interventions noted herein had limited short sleep restriction and sleep recovery periods, with no more than 7 days of recovery sleep. Despite the mixed results, these preliminary findings are encouraging and provide a basis for the potential reversibility of adverse health effects caused by sleep restriction through sleep extension. These studies have provided justification for examining sleep extension sleep, in short sleepers, as a means to provide endocrine and cardiometabolic health benefits. These investigations were undertaken to determine whether benefits would also be observed among chronic short sleepers, rather than sleep-restricted adequate sleepers, as included in the studies described above.

## Impact of sleep extension on body weight

Short-term recovery sleep studies suggest that the increase in weight and energy intake associated with sleep restriction may be ameliorated by extending sleep. Recent studies further tested this hypothesis but employed longer interventions in an outpatient setting. Logue et al. (23) performed a 12-week randomized controlled trial in overweight and obese adults, examining the effectiveness of lifestyle interventions on weight loss and sleep. The participants were randomized into 2 groups, each receiving weekly 60-min counseling sessions. Both groups received diet and exercise counseling, but the second group received additional sleep-related information starting at the week 4 session. Body weight was measured at each session and sleep quality and efficiency were assessed using the Pittsburgh Sleep Quality Index and the Sleep Timing Questionnaire, respectively, at weeks 0, 6, and 12. Food frequency questionnaires were collected at weeks 0 and 12. The results showed that both groups lost weight, but the group receiving additional sleep counseling lost more weight compared to the group only receiving diet and exercise counseling (−5% change from baseline vs. −2% change from baseline, respectively). In addition, both groups reported improved sleep efficiency, but no data regarding sleep duration were reported. These findings provide some evidence that following sleep recommendations, in addition to diet and exercise, may lead to greater weight loss than diet and exercise alone. However, given that both groups perceived improvements in sleep efficiency, it is unclear whether the sleep recommendations were instrumental in effecting greater weight loss. Indeed, there were several potential concerns with this study that should be noted: (1) the group receiving the additional sleep counseling ate significantly fewer added fats and sweets at baseline compared to the control group; when controlling for this, statistical significance disappeared; (2) retention rate was low, with approximately 54% of participants completing the study; (3) sleep was not assessed objectively and change in sleep duration as a result of the intervention could not be ascertained. These limitations decrease the impact of the study.

Another study (24) investigated the effects of behavioral counseling in combination with 2 weeks of sleep extension. Ten overweight adults, reporting sleeping <6.5 h/night at baseline, were provided a single baseline behavioral counseling session on sleep hygiene and asked to lengthen their TIB to 8.5 h/night. An additional counseling session was provided after the first week of the intervention if the study team deemed it necessary. Prescribed bedtimes and wake times were given to individuals based on their preferred schedules. Wrist actigraphy was used to objectively measure sleep throughout the study, and appetite was assessed at baseline and endpoint using visual analog scales. At the end of the 2-week intervention, participants had increased their sleep duration by an average of 1.6 h compared to baseline (7.1 vs. 5.6 h). Additionally, there was a 14% decrease in overall appetite and a 62% decrease in the desire for sweet and salty foods. These results are encouraging given that a recent meta-analysis concluded that the increase in energy intake after sleep restriction is approximately 385 kcal/d, (18) which is sufficient to lead to weight gain if sustained over time. Furthermore, the increased consumption was accompanied by a significant increase in fat intake. Although the study lacked a control group, (24) the increase in sleep duration associated with the decrease in appetite is promising and should be followed by controlled trials to determine if increasing sleep duration from short to adequate reduces food intake, improves dietary habits, and leads to improved body weight over time. It is also important to note that the study did not assess actual food intake and was only 2 weeks long, a period too short to observe effects on body weight. Therefore, unanswered questions remain as to the influence of sleep extension on body weight.

There have been several mechanisms put forth to explain the increase in energy intakes observed with sleep restriction. One proposition is that sleep restriction leads to changes in appetite-regulating hormones. Similar to the recovery sleep studies, the effects sleep restriction has on these hormones, particularly leptin, are varied (13–15). Another is that sleep restriction stimulates neuronal networks associated with reward, which increases the salience of foods (34). Despite divergent opinions on the mechanism, there is strong consensus that sleep restriction leads to increased food intake (17, 18, 35). Thus, extending sleep could potentially reverse this increase in food intake. In a study performed by Al Khatib et al. (25) participants reported a reduced intake of free sugars compared to a control group after 4 weeks of 1–1.5 h/night sleep extension. This trial investigated the feasibility of sleep extension, via behavioral counseling, to affect body weight and cardiometabolic health. Forty-one healthy adults with body mass index 18.4–24.9 kg/m^2^ who were self-reported short sleepers (5–7 h/night) were enrolled. The goal for the sleep extension group was to lengthen sleep duration by 1–1.5 h/night for 4 weeks. This was attempted by providing a single sleep consultation at baseline to design and implement a personalized strategy with prescribed bed times and wake times. There were 4 in-person visits throughout the study, 2 at baseline and 2 at endpoint, to assess outcomes related to energy balance (food intake, energy expenditure, and body composition) and cardiometabolic risk factors (blood biomarkers and BP). Wrist actigraphy was used as an objective measure of sleep duration to verify participants' adherence to sleep recommendations. At the end of the intervention, the sleep extension group had significantly increased their sleep duration by 21 min, whereas the control group reduced their sleep by 11 min. However, only 3 of 21 participants in the sleep extension group were able to reach the weekly sleep goal of 7–9 h of sleep/night. Possibly due to the minimal improvements in sleep duration in the sleep extension group, there was no significant difference in body composition or cardiometabolic risk factors between groups after the 4-week period. Additionally, relative to baseline, the sleep extension group experienced poorer sleep quality, as measured by actigraphy, compared to the control group. However, the sleep extension group reported improved perceived sleep quality compared to the control group, as reflected by a decrease in Pittsburgh Sleep Quality Index score. The decrease in sleep quality when measured objectively may have been the result of a longer TIB and may diminish over time.

Overall, these studies examining the impact of sleep extension on body weight regulation suggest that interventions including sleep education plus a prescribed sleep extension schedule have the potential to lead to improved body weight and food choices. However, several limitations hinder definitive statements and recommendations to lengthen sleep duration for weight management at this time. All of these studies are limited by their small sample size, with the intervention groups ranging from 10 to 21 subjects, and variability in participant characteristics. Additionally, while each provided some type of sleep education and counseling, the content was unique to each study and the extent of counseling (duration and intensity) varied between studies.

## Impact of sleep extension on inflammation and blood pressure

Clinical interventions have provided evidence that restricting sleep is associated with increased inflammatory markers (2) and BP (20) which are associated with cardiovascular disease (CVD). Recently, several clinical trials have examined if sleep extension could help alleviate these risks. Haack et al. (26) performed a 6-week randomized controlled trial studying 22 adults with prehypertension or type 1 hypertension who slept 7 h/night or less. After 2 weeks of actigraphy recordings to verify short sleep, participants were randomized to a sleep extension group, where they were instructed to increase their TIB by 1 h and were prescribed individualized bed times and wake times, or to a sleep maintenance group, who kept their habitual bedtimes and wake times. Both groups received instructions on how to improve sleep hygiene and, at baseline and endpoint, 24 h BP was monitored and a fasting blood sample was taken. As hypothesized, at the end of the 6-week intervention period, the sleep extension group had an increase in daily sleep duration of 35 ± 9 min. Additionally, the systolic and diastolic beat-to-beat BP average over the 24 h recording significantly decreased in the sleep extension group from baseline to endpoint by 14 ± 3 and 8 ± 3 mmHg, respectively. Decreased beat-to-beat BP variability is associated with CVD, end organ damage, and vascular elasticity (36, 37). However, there were no changes in overall BP or inflammatory markers.

McGrath et al. (27) also examined adults with elevated BP who had self-reported difficulties sleeping. Within an 8-week trial, 134 participants who were self-reported poor sleepers were randomized to either standard care or sleep intervention. The intervention group received weekly lifestyle sessions using a tool called Sleepio. Sleepio is an online platform that provides education on sleep hygiene and cognitive behavioral therapy and has been shown to improve sleep quality in patients with insomnia (38). The study investigated whether there was a difference in 24 h systolic BP, 24 h diastolic BP, peak and mean diurnal and nocturnal systolic BP and diastolic BP, and sleep quality between the two groups. At the end of the 8 weeks, the intervention group reported improved sleep quality, but there was no difference between the intervention and standard care groups in BP or other CVD-related measures. Participants did not provide data on sleep duration at baseline. Additionally, no objective measure of sleep was obtained throughout the study, nor was there a sleep extension component. For these reasons, the trial may not have produced the same improvements observed previously, (26) despite its longer intervention period and larger sample size. Although the study by McGrath et al. (27) showed no significant results regarding BP, the improved beat-to-beat BP average observed by Haack et al. (26) is encouraging and warrants further investigation into the role sleep extension may have in modulating CVD risk.

## Impact of sleep extension on glucose and insulin sensitivity

Insulin resistance is associated with numerous diseases including obesity, metabolic syndrome, and type 2 diabetes mellitus (39). Sleep may be a potential modifiable risk factor for these disorders (1). There is extensive research describing the negative effects sleep restriction on glucose levels and insulin sensitivity, (21, 22, 40) however there is much less information available on the effects of sleep extension on these outcomes. In fact, we could find only one study that investigated the effects of sleep extension on glucose and insulin (28). The goal of that study was to increase sleep duration by 1 h, every night, for 6 weeks. Actigraphy was used to verify that participants were short sleepers, sleeping <7 h/night. Participants were given instructions on proper sleep hygiene and individualized sleep schedules at the beginning of the study, but no other lifestyle intervention was included. Compliance was verified every 2 weeks during the intervention. Participants successfully extended their TIB and sleep duration throughout the intervention compared to their habitual sleep, reaching an increase of 54 ± 33 min in the first 2 weeks, 48 ± 31 min over the second 2 weeks, and 44 ± 34 min over the last 2 week. At the end of the 6-week intervention, there was a significant improvement in the insulin-to-glucose ratio, indicating lessened insulin resistance. As with other studies of this type, there was no comparison group, and there was high inter-individual variation in the amount of additional sleep that was obtained.

## Conclusion

There are limited clinical trials investigating the effects of sleep extension on health. The studies that have been conducted vary greatly in study length, participant characteristics, and intervention types. Two of the studies discussed did not use an objective measure of sleep to examine sleep quality and duration, providing only subjective data (23, 27). Additionally, while each study provided some type of sleep education, the two studies that did not objectively measure sleep were the only ones to provide continuous counseling sessions throughout the intervention period (23, 27). The other trials only included one counseling session at the start of the intervention, with the session content varying from study to study. The studies that used actigraphy to monitor sleep provided participants with specific sleep schedules, prescribing an extension in TIB of 1–1.5 h/night, (25, 26, 28) with the exception of one study that imposed a TIB of 8.5 h/night (24). It is worth noting that the trials that utilized objective measurements and individualized sleep extension interventions achieved greater benefits compared to the ones that utilized counseling sessions alone. Also, each study had different inclusion criteria, with two trials not requiring that participants be short sleepers at baseline (23, 27). Due to these study differences, it is difficult to provide recommendations regarding the impact of sleep extension on health.

Furthermore, it is unclear whether sleep quality improves as a result of sleep extension. Participants reported significant improvements of sleep quality in only one trial, (27) in which sleep was not a primary outcome and objective measures were not used. Two of the three studies that utilized objective measures of sleep reported no change in sleep quality, (26, 28) while the other (25) found that the sleep extension group experienced decreased sleep quality compared to the control group. However, this decline could be due to a period of adjustment that potentially would resolve over time and should be monitored in future trials. To that effect, it may be important for future studies to include a run-in period since Cizza et al. (41) have reported improvements in sleep duration of 15–30 min depending on measurement type, objective vs. subjective, respectively, during a waiting period between screening and randomization.

In addition to assessing sleep quality, future sleep extension studies would benefit from being performed exclusively in short sleepers, including larger sample sizes, measuring sleep duration and compliance using objective tools, and providing a prescribed sleep schedule in conjunction with counseling sessions throughout the intervention. In light of the decline in the average sleep duration among US adults, paired with the knowledge that short sleep increases the risk of obesity, CVD, and diabetes, it is encouraging to know that increasing sleep is a feasible endeavor that may be utilized in the future as a behavioral intervention to help alleviate these health burdens. However, standardized, longer-term, randomized intervention trials are needed to verify these preliminary findings.

## Author contributions

TP, BA, and M-PS-O contributed to the conception and organization of the review. TP wrote the first draft, TP and M-PS-O wrote sections of the manuscript. All authors contributed to manuscript revision, read and approved the submitted version.

### Conflict of interest statement

The authors declare that the research was conducted in the absence of any commercial or financial relationships that could be construed as a potential conflict of interest.

## Abbreviations

BP: blood pressure
CVD: cardiovascular disease
SSD: short sleep duration
TIB: time in bed.

## References

[1] St-Onge MP Grandner MA Brown D Conroy MB Jean-Louis G Coons M. Sleep duration and quality: impact on lifestyle behaviors and cardiometabolic health: a scientific statement from the American Heart Association. Circulation (2016) 134:e367–86. 10.1161/CIR.0000000000000444 27647451 PMC5567876

[2] van Leeuwen WM Lehto M Karisola P Lindholm H Luukkonen R Sallinen M. Sleep restriction increases the risk of developing cardiovascular diseases by augmenting proinflammatory responses through IL-17 and CRP. PLoS ONE (2009) 4:e4589. 10.1371/journal.pone.0004589 19240794 PMC2643002

[3] Dettoni JL Consolim-Colombo FM Drager LF Rubira MC Souza SB. Cardiovascular effects of partial sleep deprivation in healthy volunteers. J Appl Physiol. (2012) 113:232–6. 10.1152/japplphysiol.01604.2011 22539169

[4] Nedeltcheva AV Kessler L Imperial J Penev PD. Exposure to recurrent sleep restriction in the setting of high caloric intake and physical inactivity results in increased insulin resistance and reduced glucose tolerance. J Clin. Endocrinol Metab. (2009) 94:3242–50. 10.1210/jc.2009-0483 19567526 PMC2819825

[5] Patel SR Malhotra A Gottlieb DJ White DP Hu FB. Correlates of long sleep duration. Sleep (2006) 29:881–9. 10.1093/sleep/29.7.881 16895254 PMC3500381

[6] Jean-Louis G Williams NJ Sarpong D Pandey A Youngstedt S Zizi F. Associations between inadequate sleep and obesity in the US adult population: analysis of the national health interview survey (1977–2009). BMC Public Health (2014) 14:290. 10.1186/1471-2458-14-290 24678583 PMC3999886

[7] Itani O Jike M Watanabe N Kaneita Y. Short sleep duration and health outcomes: a systematic review, meta-analysis, and meta-regression. Sleep Med. (2017) 32:246–56. 10.1016/j.sleep.2016.08.006 27743803

[8] Cappuccio FP Taggart FM Kandala NB Currie A Peile E Stranges S. Meta-analysis of short sleep duration and obesity in children and adults. Sleep (2008) 31:619–26. 10.1093/sleep/31.5.619 18517032 PMC2398753

[9] St-Onge MP O'Keeffe M Roberts AL RoyChoudhury A Laferrere B. Short sleep duration, glucose dysregulation and hormonal regulation of appetite in men and women. Sleep (2012) 35:1503–10. 10.5665/sleep.2198 23115399 PMC3466797

[10] Shechter A Grandner MA St-Onge MP. The role of sleep in the control of food intake. Am J Lifestyle Med. (2014) 8:371–4. 10.1177/1559827614545315 27065757 PMC4824633

[11] Shechter A Rising R Albu JB St-Onge MP. Experimental sleep curtailment causes wake-dependent increases in 24-h energy expenditure as measured by whole-room indirect calorimetry. Am J Clin Nutr. (2013) 98:1433–9. 10.3945/ajcn.113.069427 24088722 PMC3831535

[12] Park HK Ahima RS. Physiology of leptin: energy homeostasis, neuroendocrine function and metabolism. Metab Clin Exp. (2015) 64:24–34. 10.1016/j.metabol.2014.08.004 25199978 PMC4267898

[13] Bosy-Westphal A Hinrichs S Jauch-Chara K Hitze B Later W Wilms B. Influence of partial sleep deprivation on energy balance and insulin sensitivity in healthy women. Obes Facts (2008) 1:266–73. 10.1159/000158874 20054188 PMC6515888

[14] Buxton OM Cain SW O'Connor SP Porter JH Duffy JF Wang W. Adverse metabolic consequences in humans of prolonged sleep restriction combined with circadian disruption. Sci Transl Med. (2012) 4:129ra43. 10.1126/scitranslmed.3003200 22496545 PMC3678519

[15] St-Onge MP. The role of sleep duration in the regulation of energy balance: effects on energy intakes and expenditure. J Clin Sleep Med. (2013) 9:73–80. 10.5664/jcsm.2348 23319909 PMC3525993

[16] Spaeth AM Dinges DF Goel N. effects of experimental sleep restriction on weight gain, caloric intake, and meal timing in healthy adults. Sleep (2013) 36:981–90. 10.5665/sleep.2792 23814334 PMC3669080

[17] St-Onge MP Roberts AL Chen J Kelleman M O'Keeffe M RoyChoudhury A Short sleep duration increases energy intakes but does not change energy expenditure in normal-weight individuals. Am J Clin Nutr. (2011) 94:410–6. 10.3945/ajcn.111.013904 21715510 PMC3142720

[18] Al Khatib HK Harding SV Darzi J Pot GK. The effects of partial sleep deprivation on energy balance: a systematic review and meta-analysis. Eur J Clin Nutr. (2017) 71:614–24. 10.1038/ejcn.2016.201 27804960

[19] Kaptoge S Seshasai SR Gao P Freitag DF Butterworth AS Borglykke A. Inflammatory cytokines and risk of coronary heart disease: new prospective study and updated meta-analysis. Eur Heart J. (2014) 35:578–89. 10.1093/eurheartj/eht367 24026779 PMC3938862

[20] Robillard R Lanfranchi PA Prince F Filipini D Carrier J. Sleep deprivation increases blood pressure in healthy normotensive elderly and attenuates the blood pressure response to orthostatic challenge. Sleep (2011) 34:335–9. 10.1093/sleep/34.3.335 21358850 PMC3041709

[21] Broussard JL Ehrmann DA Van Cauter E Tasali E Brady MJ. Impaired insulin signaling in human adipocytes after experimental sleep restriction: a randomized, crossover study. Ann Int Med. (2012) 157:549–57. 10.7326/0003-4819-157-8-201210160-00005 23070488 PMC4435718

[22] Rao MN Neylan TC Grunfeld C Mulligan K Schambelan M Schwarz JM. Subchronic sleep restriction causes tissue-specific insulin resistance. J Clin Endocrinol Metab. (2015) 100:1664–71. 10.1210/jc.2014-3911 25658017 PMC4399283

[23] Logue EE Bourguet CC Palmieri PA Scott ED Matthews BA Dudley P. The better weight-better sleep study: a pilot intervention in primary care. Am J Health Behav. (2012) 36:319–34. 10.5993/AJHB.36.3.4 22370434

[24] Tasali E Chapotot F Wroblewski K Schoeller D. The effects of extended bedtimes on sleep duration and food desire in overweight young adults: a home-based intervention. Appetite (2014) 80:220–4. 10.1016/j.appet.2014.05.021 24858836 PMC4112413

[25] Al Khatib HK Hall WL Creedon A Ooi E Masri T McGowan L. Sleep extension is a feasible lifestyle intervention in free-living adults who are habitually short sleepers: a potential strategy for decreasing intake of free sugars? A randomized controlled pilot study. Am J Clin Nutr. (2018) 107:43–53. 10.1093/ajcn/nqx030 29381788 PMC5972593

[26] Haack M Serrador J Cohen D Simpson N Meier-Ewert H Mullington JM. Increasing sleep duration to lower beat-to-beat blood pressure: a pilot study. J Sleep Res. (2013) 22:295–304. 10.1111/jsr.12011 23171375 PMC3582793

[27] McGrath ER Espie CA Power AM Newell AW Kelly J Duffy C. Sleep to lower elevated blood pressure: a randomized controlled trial (SLEPT). Am J Hypertens. (2017) 30:319–27. 10.1093/ajh/hpw132 28391289

[28] Leproult R Deliens G Gilson M Peigneux P. Beneficial impact of sleep extension on fasting insulin sensitivity in adults with habitual sleep restriction. Sleep 38:707–15. 10.5665/sleep.4660 25348128 PMC4402666

[29] National Sleep Foundation (2013) International Bedroom Poll. Arlington, TX: National Sleep Foundation WD.

[30] van Leeuwen WM Hublin C Sallinen M Harma M Hirvonen A Porkka-Heiskanen T. Prolonged sleep restriction affects glucose metabolism in healthy young men. Int J Endocrinol. (2010) 2010:108641. 10.1155/2010/108641 20414467 PMC2857625

[31] Faraut B Boudjeltia KZ Dyzma M Rousseau A David E Stenuit P. Benefits of napping and an extended duration of recovery sleep on alertness and immune cells after acute sleep restriction. Brain Behav Immun. (2011) 25:16–24. 10.1016/j.bbi.2010.08.001 20699115

[32] Spiegel K Leproult RM L'Hermite-Baleriaux Copinschi G Penev PD Van Cauter E. Leptin levels are dependent on sleep duration: relationships with sympathovagal balance, carbohydrate regulation, cortisol, and thyrotropin. J Clin Endocrinol Metab. (2004) 89:5762–71. 10.1210/jc.2004-1003 15531540

[33] Pejovic S Basta M Vgontzas AN Kritikou I Shaffer ML Tsaoussoglou M. Effects of recovery sleep after one work week of mild sleep restriction on interleukin-6 and cortisol secretion and daytime sleepiness and performance. Am J Physiol Endocrinol Metab. (2013) 305:E890–6. 10.1152/ajpendo.00301.2013 23941878 PMC3798707

[34] Chaput JP St-Onge MP. Increased food intake by insufficient sleep in humans: are we jumping the gun on the hormonal explanation? Front Endocrinol. (2014) 5:116. 10.3389/fendo.2014.00116 25076940 PMC4098122

[35] Nedeltcheva AV Kilkus JM Imperial J Kasza K Schoeller DA Penev PD. Sleep curtailment is accompanied by increased intake of calories from snacks. Am J Clin Nutr. (2009) 89:126–33. 10.3945/ajcn.2008.26574 19056602 PMC2615460

[36] Gershon RC Cook KF Mungas D Manly JJ Slotkin J Beaumont JL. Language measures of the NIH Toolbox Cognition Battery. J Int Neuropsychol Soc. (2014) 20:642–51. 10.1017/S1355617714000411 24960128 PMC4558909

[37] Xia Y Wu D Gao Z Liu X Chen Q Ren L. Association between beat-to-beat blood pressure variability and vascular elasticity in normal young adults during the cold pressor test. Medicine (2017) 96:e6000. 10.1097/MD.0000000000006000 28225488 PMC5348138

[38] Espie CA Kyle SD Williams C Ong JC Douglas NJ Hames P. A randomized, placebo-controlled trial of online cognitive behavioral therapy for chronic insomnia disorder delivered via an automated media-rich web application. Sleep (2012) 35:769–81. 10.5665/sleep.1872 22654196 PMC3353040

[39] Ferrannini E Natali A Bell P Cavallo-Perin P Lalic N Mingrone G. Insulin resistance and hypersecretion in obesity. European Group for the Study of Insulin Resistance (EGIR). J Clin Investig. (1997) 100:1166–73. 10.1172/JCI119628 9303923 PMC508292

[40] Rafalson L Donahue RP Stranges S Lamonte MJ Dmochowski J Dorn J. Short sleep duration is associated with the development of impaired fasting glucose: the Western New York health study. Ann Epidemiol. (2010) 20:883–9. 10.1016/j.annepidem.2010.05.002 20620078 PMC2962429

[41] Cizza G Piaggi P Rother KI Csako G Sleep Extension Study Group. Hawthorne effect with transient behavioral and biochemical changes in a randomized controlled sleep extension trial of chronically short-sleeping obese adults: implications for the design and interpretation of clinical studies. PLoS ONE (2014) 9:e104176. 10.1371/journal.pone.0104176 25141012 PMC4139265

